# In Vitro Antibacterial, Antifungal, Nematocidal and Growth Promoting Activities of *Trichoderma hamatum* FB10 and Its Secondary Metabolites

**DOI:** 10.3390/jof7050331

**Published:** 2021-04-24

**Authors:** Alaa Baazeem, Abdulaziz Almanea, Palanisamy Manikandan, Mohammed Alorabi, Ponnuswamy Vijayaraghavan, Ahmed Abdel-Hadi

**Affiliations:** 1 Department of Biology, College of Science, Taif University, P.O. Box 11099, Taif 21944, Saudi Arabia; aabaazeem@tu.edu.sa; 2 Section of Microbiology, Department of Laboratory, King Saud Hospital, Unaizah 51911, Saudi Arabia; aalmanea@moh.gov.sa; 3 Department of Medical Laboratory Sciences, College of Applied Medical Sciences, Majmmah University, Majmaah 11952, Saudi Arabia; m.palanisamy@mu.edu.sa; 4 Greenlink Analytical and Research Laboratory (India) Private Limited, Coimbatore 641014, Tamil Nadu, India; 5 Department of Biotechnology, College of Science, Taif University, P.O. Box 11099, Taif 21944, Saudi Arabia; morabi@tu.edu.sa; 6 Bioprocess Engineering Division, Smykon Biotech, Nagercoil, Kanyakumari 629201, Tamil Nadu, India; 7 Botany and Microbiology Department, Faculty of Science, Al-Azhar University, Assiut Branch, 71524 Assiut, Egypt

**Keywords:** *Trichoderma hamatum* FB10, antibacterial activity, antifungal activity, cell free extract, volatile compounds, biological control, soil enzymes

## Abstract

Microbial natural biocides have attracted much more attention in recent years in order to avoid the unrestricted use of chemical biocides in the environment. The aim of this study is to analyze the antibacterial and antifungal activities of secondary metabolites and growth promoting, nematicidal, and soil enzyme activity mediated by *Trichoderma hamatum* FB10. The bactericidal and fungicidal activities were performed using cell-free extract. Results revealed that the selected strain exert antibacterial activity against *Acidovorax avenae*, *Erutimacarafavora*, and *Xanthomonas campestris*. The selected fungal strain FB10 showed antagonistic activity against fungal pathogens such as, *S. sclerotiorum*, *Rhizoctonia solani*, *Alternaria radicina*, *Alternaria citri*, and *Alternaria dauci*. Among the bacterial pathogens, *A. avenae* showed least MIC (30 ± 2.5 µg/mL) and MBC (70 ± 1.25 µg/mL) values. *T. hamatum* FB10 strain synthesized bioactive volatile secondary metabolite, which effectively inhibited the growth of bacteria and fungi and indicated the presence of 6-pentyl-alpha-pyrone as the major compound (67.05%). The secondary metabolite synthesized by *T. hamatum* FB10 showed nematicidal activity against *M. incognita* eggs. Egg hatch inhibition was 78 ± 2.6% and juvenile stage mortality rate was 89 ± 2.5% when the strain FB10 was treated with nematode. The cell free extract of *T. hamatum* FB10 showed protease, amylase, cellulase, chitinase, glucanase activities. *T. hamatum* FB10 inoculated with green gram increased 11% plant height, compared to the control. The fresh weight of the experimental group inoculated with *T. hamatum* FB10 increased 33.6% more compared to the control group. The green gram seedlings inoculated with *T. hamatum* FB10 increased 18% more dry weight than control group. Soil enzymes such as, urease, phosphatase, catalase and saccharase were improved in the soil inoculated with *T. hamatum* FB10. These biochemical components play potent role in soil fertility, energy conversion, and in soil organic matter conversion.

## 1. Introduction

Most of the fungicides are synthetic pesticides widely applied in forests, archeological, parks, and agriculture areas. Pesticide pollution, mainly of water and soil, due to unrestricted use of these synthetic chemicals has prompted the search for alternate eco-friendly substances based on natural sources. These natural sources avoid contamination of water and environment and minimize the risk to animal and human health [1,2,3]. Microorganisms, algae, lichens and plants are producers of various secondary metabolites with novel biological properties, such as, antifungal, antibacterial, antiplasmodium, cytotoxic, aggregation, antiplatelet, immunostimulant, enzyme-inhibiting, algicide, antitumor, anticancer, antiviral, and phytotoxic activities [4]. Several secondary metabolites with novel antibacterial and antifungal activities have been characterized from various fungal sources [5,6]. These secondary metabolites derived from natural sources have various structural classes of compounds (steroids, terpenes, poliketides, anthraquniones, and alkaloids) and most of them show unique action to overcome various drug resistances [7,8,9,10]. The biological control of various plant diseases, microorganisms, nematodes and insects has been reported as a supplement to chemical control or an alternative to chemical pesticides [11]. The biocontrol property of various microorganisms is exerted either indirectly eliciting a plant-mediated resistance response or through antagonism of pathogen development [12]. Mechanisms responsible for antifungal activity include mycophagy, parasitism, competition for nutrients and minerals, and competition for colonization [13]. It is an important mechanism of bacterial antagonists to affect the plant pathogens by the secretion of extracellular antimicrobial secondary metabolites. The well-known antibiotic substances are bio-surfactants, toxins and antibiotics [14]. In recent years, it was stated that terrene derivatives, terrenes, ester, ethers, aldehydes, ketenes, alcohols, hydrocarbons, and various heteroaromatic substances synthesized by various bacteria can effectively influence the growth of fungal strains [15,16,17]. Secondary metabolites produced by microorganisms caused an effective inhibition of the germination of conidia in various pathogenic fungi [18]. Recent experiments revealed the inhibitory role of secondary metabolites from microorganisms, including in vitro inhibition for the germination of conida of *Penicillium expansum* and *Penicillium digitatum* [19]. The phytopathogen, *Burkholderia gladioli* had the ability to produce various hydrolytic enzymes, such as glucanase, amylase, cellulase, protease, and chitinase, that may have a potential effect on the growth of fungi [20]. Among these hydrolytic enzymes, chitinases have tremendous application in industrial and pharmaceutical fields. These enzymes degrade chitin in chitooligosaccharides. In a study, Jijakli and Lepoivre [21] showed that in vitro and in vivo antifunal activity of *Pichia anomala* are mainly based on the biosynthesis of β-1,3-exoglucanase enzyme. Saligkarias et al. [22] reported the influence of extracellular protease synthesized by the phytopathogens with novel in vitro and in vivo application against fungi. In recent years, various biocontrol agents are reported including, bacterial, such as *Pseudomonas*, *Bacillus*, *Agrobacterium*, and fungi, such, as, *Trichoderma*, *Pseudozyma*, *Gliocadium*, *Coniothyrium*, *Candida*, *Aspergillus*, and metabolites from *Ampelomyces* [23]. Some of the fungi, including *Trichoderma* sp., have the ability to survive in extreme environment and proved effective in the management of various crop diseases caused by several fungal species. Fungi proved effective in the crop management and to control fungal diseases. *Trichoderma* spp. have been isolated from the soil samples mostly from the rhizosphere of the leguminous plants. These fungi act as antagonists and parasites of various phytopathogenic fungi and avirulent plant symbionts, thus effectively protecting plants from various diseases [24]. The antagonistic activities of fungi from the genus *Trichoderma* are based on the activation of direct and indirect mechanisms. The direct mechanisms are the production of lytic enzymes, active metabolites, and mycoparasitism, whereas indirect mechanisms are competition for nutrients and space, induction of plant defenses, and promotion of growth. These direct and indirect mechanisms depending on strain and species and can act synergistically [25]. *Trichoderma* species produce various secondary metabolites able to inhibit the growth of several bacterial and fungal pathogens. The volatile compounds synthesized by the fungal strains diffused in the soil medium and preventing the physiology of pathogenic microorganisms [26].

## 2. Materials and Methods

### 2.1. Characterization of Trichoderma Strains

The fungal strain of FB10 was characterized as *Trichoderma hamatum* FB10 based on morphological and biochemical characters and 18S *rDNA* gene sequencing. The root tissues were taken from tomato plant and washed with sodium 1% hypochloride solution for 3 min. It was rinsed with sterile double distilled water and transferred onto potato agar plates (PDA). Streptomycin sulphate was incorporated with the culture medium to inhibit the growth of bacteria. The culture plates were incubated for 72 h for 25 ± 2 °C. Pure fungal colonies were subcultured on PDA medium. For the identification of the fungal strain, the ITS region of *rDNA* was performed. Amplification was performed using Taq DNA polymerase and universal forward and reverse primers. The amplified DNA was sequenced, and sequences were submitted in Genbank Database.

### 2.2. Production of Secondary Metabolites

*T. hamatum* FB10 was inoculated into 1 L of sterile potato dextrose broth medium. The culture was incubated for 10–15 days at 28 ± 2 °C under shaking (150 rpm/min). The culture was filtered using a vacuum filtration unit using Whatman no 1 filter paper. The culture filtrates were maintained at −70 °C.

### 2.3. Extraction of Secondary Metabolites

The 10- and 15-day culture extracts of *T. hamatum* FB10 were extracted using ethyl acetate for 4 times. The final concentration of extract and solvent was 1:1 ratio. The organic fraction of the solvent fraction was dried under reduced pressure at 28 ± 2 °C. The residues obtained were suspended in methyl alcohol for the determination of secondary metabolites. Dimethyl sulfoxide (DMSO) (10%) was added with the sample and used for the determination of antimicrobial properties.

### 2.4. Antimicrobial Activity

The qualitative antifungal assay of the methanol fraction of *T. hamatum* FB10 was performed by disc diffusion method as described previously. To determine antagonistic activity of the culture supernatant against fungi, the fungal pathogens such as, *S. sclerotiorum*, *Rhizoctonia solani*, *Alternaria radicina*, *Alternaria citri*, and *Alternaria dauci* were selected. The antibacterial activity of the extract was assayed by well diffusion method. The bacterial pathogens such as, *Xanthomonas citri*, *Xanthomonas campestris*, *Erutima carafavora*, *Clavibacter michiganensis* and *Acidovorax avenae* were used. This experiment was carried out using pathogenic strains at 37 °C and the cell density was maintained appropriately (10^5^ CFU/mL) using sterile double distilled water. The diluted fungal strains were inoculated on MHA and PDA medium, and the sample was impregnated and dried appropriately using a Whatman paper disc (6 mm). The culture plate was left for 60 min for diffusion of sample and the plate was incubated for 48 h at 28 ± 2 °C. Carbendazine was used as the positive control for fungi and ampicillin was used as the control for bacteria. The experiment was performed in duplicate, and the average values were considered for statistical analysis.

### 2.5. Minimal Inhibitory Concentration Determination

The minimal inhibitory concentration (MIC) was determined using broth dilution method. Experiments were performed in MH broth (Himedia, Mumbai, India) medium supplied with Tween 80 (5%). The selected bacterial strains were cultured at 37 °C in Mueller Hinton broth (MH) broth medium. Then, different concentrations of diluted culture supernatant were added and incubated the culture (10^5^ CFU/mL) for 24 h at 28 ± 2 °C. Standard drugs, ciprofloxacin (antibacterial agent) and nystatin (antifungal agent) were prepared at 1–500 µg/mL concentration. The MIC value was defined as the extreme lowest concentration of the secondary metabolites controlling visible growth. Then the culture (10 µL) was transferred to nutrient agar plates and the complete growth inhibition was considered as minimum bactericidal concentrations (MBC).

### 2.6. GC-MS Analysis of Secondary Metabolites

The volatile compounds of secondary metabolites from the crude ethyl acetate extract of fungi were determined using a gas chromatography (Agilent technologies). About 1 µL sample was injected in split mode and the flow of the carrier gas was adjusted to 1 mL/min. The injector temperature was maintained as 250 °C, whereas the detector temperature was 280 °C. The oven temperature was initially adjusted at 50 °C for 5 min and increased to 260 °C and held for 5 min. NIST database was used to find the compound of mass spectrum separated during analyses.

### 2.7. Nematicidal Property

The fugal strain was cultured in Potato Dextrose broth, Carrot broth, Cornmeal broth, Modified Potato Dextrose broth and water broth and nematicidal property was determined. Anti-nematode property was tested in two different analyses on second stage juvenile of *Meloidogyne incognita* and eggs. Secondary metabolites extracted using ethyl acetate was suspended in ethyl acetate at the final concentration of 100 mg/mL. *M. incognita* eggs were counted, and 100 were maintained in a 0.6-mL round bottom microtitre plate. It was exposed to secondary metabolites in microtitre plates. In another experiment, with 0.6 mL sterilized water, 100 second stage juvenile *M. incognita* were incubated with 100 mg/mL extract. The plates were incubated for 72 h and the mortality rate was recorded.

### 2.8. Hydrolytic Enzyme Production by the Fungal Strain

Protease activity of the culture filtrate was tested according to the methods of Vijayaraghavan and Vincent [27] using casein as a substrate. Chitinase activity of the filtrate was tested using 1% colloidal chitin. Cellulase and amylase activities were analyzed using carboxymethyl cellulose (0.5%) and soluble starch (1%) as a substrate [28]. Polygalacturanase and pectinase activities were assayed using polygalacturanic acid (0.5%) and pectin (1%) substrate. Enzyme activities were expressed as IU/mL enzyme.

### 2.9. Analysis of T. hamatum FB10 on Growth Promoting Activity in Plants

*T. hamatum* FB10 was cultured in potato dextrose broth (pH 6.0) and incubated for eight days under agitation at 28 ± 1 °C. This culture was used to inoculate the maize, cowpea, small millet, green gram and black gram seedlings. The seeds were dried under shade for about 10 days and used for this experiment. All selected seeds were surface sterilized with 0.5% (*v*/*v*) potassium permanganate for 30 min. It was further washed with sterile distilled water for three times. It was then allowed for germination on moistened gauge for four to five days. Then it was carefully transferred to a small pot (25 seeds per pot) filled with sand with adequate organic nutrients. It was maintained under greenhouse conditions and watered appropriately for 30 days before fungus culture was further inoculated. To the control only PD broth was inoculated, to the experimental pots, *T. hamatum* FB10 was inoculated. The fungal inoculation was done by transferring 50 mL of fungal broth medium into the planting hole. About 50 mL sterile broth was inoculated into the planting hole of the control plants. All plants were maintained in green house conditions and maintained for 3 months. Then, plant height, wet weight, and dry weight were analyzed.

### 2.10. Analysis of Enzymes in the Soil

Soil samples were subjected for the analysis of urease, phosphatase, catalase and saccharase. About 100 g soil samples in each treatment was sieved using 1.0–1.5 mm sieve and extracted with double distilled water. The extracted sample was used for the determination of enzymes. Enzyme activity is expressed as IU/g soil.

## 3. Results

### 3.1. Identifications of Secondary Metabolites from the Crude Ethyl Acetate Extract

Crude methanol extract of *T. hamatum* FB10 isolated was applied for the identification of secondary metabolites using GC-MS analysis. The determined spectra were analyzed using NIST standard patterns. The chromatogram of GC analysis revealed the presence of various compounds they were identified based on the retention time, peak area, and molecular formula (Table 1). The present finding revealed the presence of various compounds with antimicrobial and anticancer potentials. The major compound was identified as 6-pentyl-alpha-pyrone and detected at 22.03 min. This is one of the important bioactive compounds from the strain FB10.

### 3.2. Antimicrobial Activity of Crude Extract against Phytopathogens

**Figure 1:**
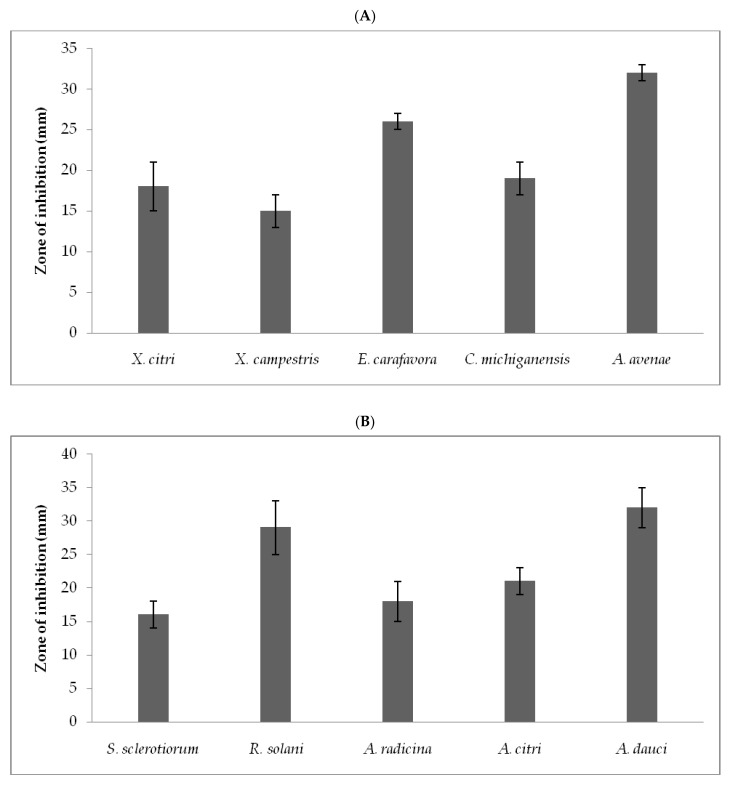
Antibacterial (**A**) and antifungal (**B**) activity of *T. hamatum* FB10 culture supernatant against pathogenic organisms. Zone of inhibition was expressed as mm diameter.

The crude extract was analyzed for its antibacterial activity against *X. citri*, *X. campestris*, *E. carafavora*, *C. michiganensis*, and *A. avenae*. The crude extract showed significant antibacterial activity against *A. avenae* (32 ± 1 mm), *E. carafavora* (26 ± 1 mm), and least activity (15 ± 2 mm) against *X. campestris*. The selected fungal strains showed antagonistic activity against fungal pathogens such as *S. sclerotiorum*, *R. solani, A. radicina*, *A. citri*, and *A. dauci*. Among the fungal pathogens, *A. avenae* showed the lowest MIC (30 ± 2.5 µg/mL) and MBC (70 ± 1.25 µg/mL) values. Moreover, MIC value was within 75 µg/mL concentration for bacterial species and MBC value was less than 125 5 µg/mL concentration for the fungal strains. The fungal extract showed least activity against *X. citri* and the MIC and MBC values were 63 ± 2.1 µg/mL and 82.5 ± 2.5 µg/mL, respectively. Ciprofloxacin and nystatin were prepared at 1–500 µg/mL concentration and antibacterial activity was compared (Figure 1A,B; Table 2).

### 3.3. Nematicidal Activity of Secondary Metabolites of T. hamatum FB10

**Figure 2:**
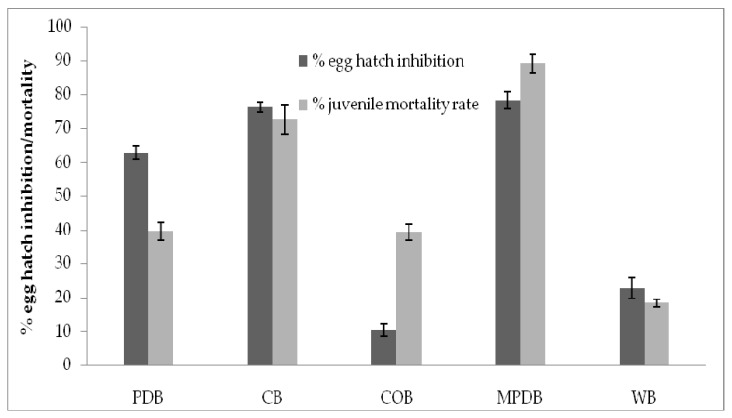
Effect of secondary metabolites from *T. hamatum* FB10 cultured in various culture media and nematicidal activity (PDB: Potato Dextrose broth; CB: Carrot broth; COB: Cornmeal broth; MPD: Modified Potato Dextrose broth; WB: Water broth).

The present finding of nematicidal activity of secondary metabolites revealed that *T. hamatum* FB10 has lot of potential to inhibit the hatching ability of *M. incognita* eggs. Egg hatch inhibition was 78 ± 2.6% when the fungal strain was cultured in modified potato dextrose broth. In the case of juvenile stage of *T. hamatum* FB10 also mortality rate was observed. The mortality rate was 89 ± 2.5% when the strain was cultured in modified potato dextrose broth. Carrot broth also showed 72.5 ± 4.4% mortality, whereas cornmeal broth showed least egg hatch inhibition activity and mortality inducing ability in juveniles (Figure 2).

### 3.4. Extracellular Enzyme Activities

The cell free extract of *T. hamatum* FB10 showed protease, amylase, cellulase, chitinase, and glucanase activities. Protease, glucanase, and chitinase activities were maximum in the culture filtrate. Moreover, the selected fungal strain FB10 showed least pectinase, amylase, cellulase and polygalacturonase activities. Hydrolytic enzymes and their activities are described in Table 3.

### 3.5. T. hamatum FB10 and Its Growth Promoting Activity

**Figure 3:**
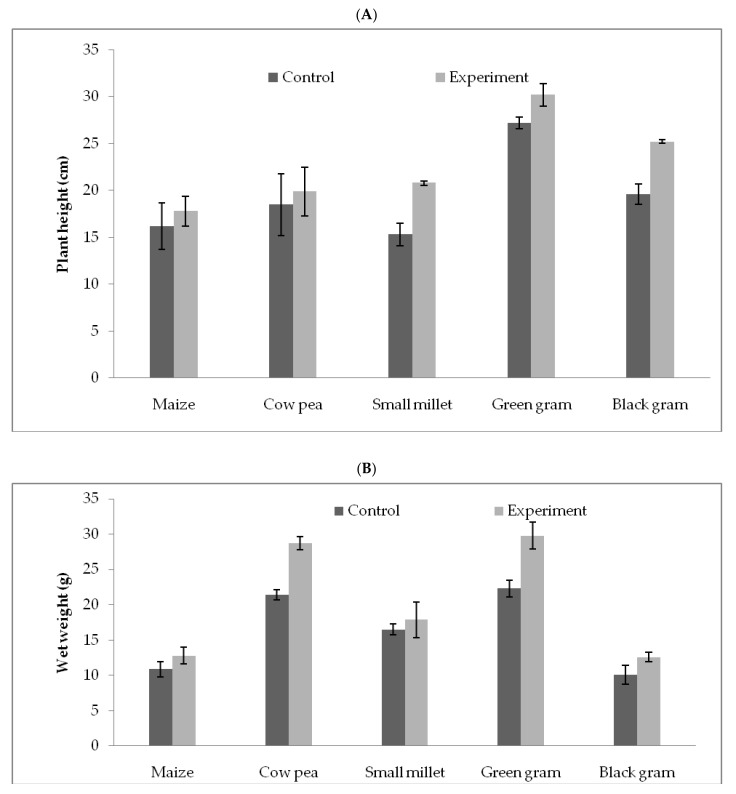

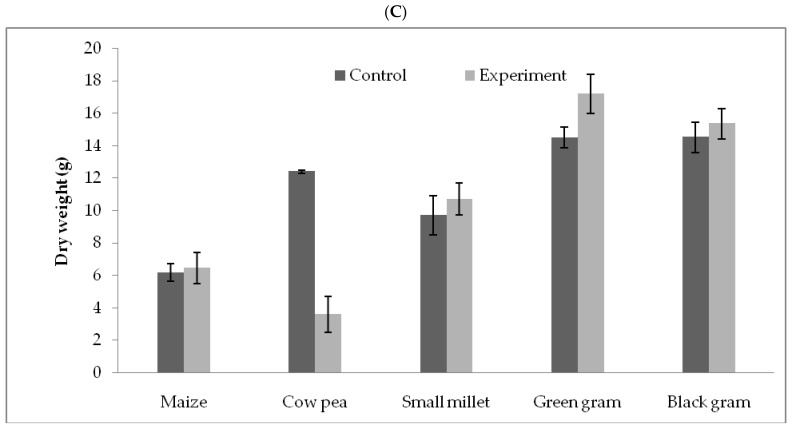
Effect of *T. hamatum* FB10 inoculated with maize, cowpea, small millet, green gram and black gram seedlings. Plant height (**A**), wet weight (**B**) and (**C**) dry weight.

*T. hamatum* FB10 was inoculated to the maize, cowpea, small millet, green gram and black gram seedlings. *T. hamatum* FB10 inoculated with green gram increased 11% plant height, compared to the control. The fresh weight of the experimental group inoculated with *T. hamatum* FB10 increased 33.6% more compared to the control group. The green gram seedlings inoculated with *T. hamatum* FB10 increased 18% more dry weight than control group. The variations in plant height, fresh weight, and dry weight in the treatment and control groups are described in Figure 3.

### 3.6. Influence of T. hamatum FB10 on Enzyme Activity of Soil

Soil enzymes, such as urease, phosphatase, catalase, and saccharase, are derived from the production of various soil microorganisms, including, bacteria, fungi and actinomycetes, animals and plants. These biochemical components play potent role in soil fertility, energy conversion and in soil organic matter conversion. Urease activity was 1.32 ± 0.26 IU/g in maize and a lower amount of urease activity was detected in rhizosphere soil associated with green gram (0.04 ± 0.1 IU/g). Phosphatase activity was 4.81 ± 0.46 IU/g in green gram soil and a decreased level was detected in small millet (1.29 ± 0.12 IU/g). Likewise, saccharase activity was maximum (2.617 ± 0.43 IU/g) in green gram soil and was low in the rhizosphere soil from cow pea (0.28 ± 0.04 IU/g).

## 4. Discussion

The healthy plants showed the presence of various endophytic bacteria and fungi. Many studies have been carried out to analyze bioactivity and biodiversity of bacterial and fungal endophytic organisms associated with various host plant species [29,30,31]. The present investigation revealed the bioactivity of *T. hamatum* FB10 isolated from the root of tomato plant. The isolated fungal species were characterized, and secondary metabolites were produced in liquid culture medium in shake flask culture. The present finding indicated that the selected *T. hamatum* FB10 exhibit potent antimicrobial activity. The ethyl acetate extract of crude sample from the endophytic strain FB10 showed potent activity than other solvent extract (methanol and chloroform). Fungi from the genus *Trichoderma* produced various secondary metabolites with robust antibiotic and antifungal activities [32]. The bioactivity of the sample extracted with ethyl acetate secondary metabolites from *T. hamatum* FB10 was highly effective compared to the other extract. In a study, Abdulmyanova et al. [33] analyzed crude ethyl acetate fractions of endophytes from *V. erecta* and *V. minor*. They showed that the ethyl acetate extracts have potent metabolites with novel biological properties. The present result might clearly indicate the availability of various compounds in *T. hamatum* FB10 that make it attack or compete antagonistic cells in a microbial population within various environmental niches. This may suggest that the *T. hamatum* FB10 has protective, selective and evolutionary roles when living inside of the plants. Trichothecenes have been reported from the fungi. These comprise various sesquiterpenes, which commonly have 12, 13-epoxy-trichothec-9-ene moiety. Moreover, the nucleus of this sesquiterpene was previously reported the fungal culture containing *Trichoderma*, *Stachyobotrs*, *Myrothecium* and *Fusarium* [34]. Macrocyclic trichothecenes isolated from the fungal species showed antimalarial, antiviral, antibacterial and antifungal, and insecticidal activities [35,36]. Yang et al. [37] isolated a fungicidal compound, 4b-hydroxy-12, 13-epoxytrichothec-9-ene. This toxin has various mechanisms of action, including inhibition of protein synthesis by disrupting the activity of peptidyl transferase. Trichodermin is an important compound determined frequently from the culture extract of *Trichoderma* species, such as, *T. koningiopsis*, *T. harzianum*, *T. longibrachiatum*, *T. viride*, and *T. brevicompactum* [36,37,38]. The volatile substances synthesized by the fungal strains diffuse into the surrounding environment and this favours the interactions between their living environment and filamentous fungi. The secondary metabolites produced by the fungal strains have broken down into various classes of antifungal compounds and contributes to antifungal activity [39]. Moreover, some endophytic fungi improved survival rate of host plants in certain environmental habitats. In fungi, 6-pentyl-alpha-pyronein was considered as one of the important secondary metabolites and it has been previously detected from various *Trichoderma* species, including, *T. koningii*, *T. harzianum*, *T. atroviride*, and *T. viride* [40]. However, the production of secondary metabolites by the endophytic strains showed a relationship with the biological property of the organism [41]. *T. hamatum* FB10 characterized in this study showed the presence of various hydrocarbons and free fatty acids. Fatty acids are organic acids with antifungal and antibacterial activities. The unsaturated fatty acids such as, butanoic acid, hexadecanoic acid and ethanolic acid were determined from the crude ethyl acetate extract of *T. hamatum* FB10. The hydrocarbon compound, hexadecane and butyrolactone were detected in GC-MS analysis. Analysis of endophytes diversity have determined relationships among host plants as well as endophytic fungi, by determining for various secondary metabolites biosynthesized from the culture extract of endophytic fungi. Endophytic *T. hamatum* FB10 was shown to yield various bioactive metabolites with antifungal and antibacterial activities. In the present study, the metabolites secreted by the fungus towards various other phytopathogens were analyzed. The anti-plant pathogenic bactericidal property of metabolites produced by the fungus has been described previously [42]. Penicillic acid is one of the fungal toxins reported previously has the potential against various pathogenic microorganisms. *Trichoderma* species isolated from the environment have the ability to stimulate the growth of bacterial and fungal pathogens. *Trichoderma* sp. uses the biocontrol mechanism such as, antibiosis, mycoparasitism and competition. These metabolic processes are mainly stimulated by the biosynthesis of specific metabolites, lytic enzymes, siderophores, antibiotics, and plant growth regulators. These organisms also synthesized chitinolytic enzymes which degraded the spores of fungi and cell walls of hyphae [43,44,45,46]. Many authors have described the antagonistic activity of strains of *Trichoderma* against various pathogenic fungal phytopathogens [47,48,49]. Fungi such as *Trichoderma harzianum* T-22, *T. harzianum*, *T. atroviride*, and *T. longibrachiatum* have shown the potential to inhibit the production of mycotoxins from *Fusarium* sp. The antifungal activity of *T. harzianum* T-22 has been determined against phytopathogenic fungi, such as *Rhizoctonia solani*, *Sclerotinia sclerotiorum*, and *Alternaria alternate* [48,49,50]. Fungi have the ability to produce secondary metabolites based on the substrate and environmental factors. Presence of suitable medium to produce bioactive secondary metabolites is very important because it supports the production of metabolites. Our finding revealed that different culture media showed variations in the production of secondary metabolites and fungal growth. It was previously reported the yield of secondary metabolites and activity when it was cultured in different growth media. *Trichoderma* is one of the prominent genera among endophytic fungi. The selected endophytic strain FB10 exhibited antifungal and antibacterial activity on selected bacterial and fungal strains. The present finding indicates that the endophyte from tomato plant had antifungal and antibacterial activities, revealing that endophytes isolated from the tomato plant are a potential source of bioactive metabolites. Endophytes are good source of various compounds with novel biological properties as observed by the antifungal and antibacterial activities of endophytes from Solanaceae. Therefore, endophytes from Solanaceae are promising sources of natural and new lead molecules that provide a potential for future research. The culture supernatant of *T. hamatum* FB10 showed maximum amount of protease, glucanase and chitinase activities. The presence of glucanase and chitinase could effectively affect the growth of phytopathogenic fungal strains by direct degradation of cell wall of fungi. Proteolytic enzymes directly degrade the cell wall of fungi, because the skeletal components of the fungal species are embedded in a matrix protein. Hence, protease activity is important for the degradation of the entire fungal cells [51,52,53]. Our results revealed that *T. hamatum* FB10 inoculated with maize, cowpea, small millet, green gram, and black gram seedlings improved plant growth. *Trichoderma* sp. improving plant growth, improving seed growth and recycling, decomposing and utilizing various soil nutrients and improved crop yields in various plants, including tomato and cotton [54,55]. *T. hamatum* FB10 inoculated pot showed improved enzyme activity than the control group. When compared with the control pot, the urease, phosphatase, catalase, and saccharase activity of rhizosphere soil improved by 12%, 291%, 27.5%, and 69%, respectively. These findings indicated that the pot treated with *T. hamatum* FB10 played a potent role in promoting enzyme mediated nutrient recycling activity. The interactions between the soil microorganisms and rhizosphere play a potent role in energy conversion, nutrient cycling, plant yield, plant growth, and energy conversion in the soil at various levels. A mixture of *Trichoderma* and organic fertilizer effectively improved crop yield and plant growth [56]. Fungi from the genus *Trichoderma* utilized organic fertilizer as a medium for the production of various plant growth promoters including indole acetic acid [57].

## 5. Conclusions

The present finding revealed a positive correlation between the biological control of the selected strain *Trichoderma hamatum* FB10 and secondary metabolites. The secondary metabolites and the volatile compounds synthesized by the strain FB10 influenced antibacterial, antifungal, and nematocidal activity. The strain FB10 induced the growth of maize, cowpea, small millet, green gram, and black gram seedlings. Soil enzyme activity, such as that of urease, phosphatase, catalase, and saccharase, was improved. These enzymes play potent roles in soil fertility, energy conversion, and in soil organic matter conversion.

## Figures and Tables

**Table 1:** GC-MS analysis of the ethyl acetate extract of the volatile compounds produced by *T. hamatum* FB10.

Peak No	Chemical Name	Chemical Formula	Retention Time (min)	Abundance (%)
1	Butyrolactone	C_4_H_6_O_2_	7.371	63.51
2	Sulfurous acid, octyl 2-pentyl ester	C_13_H_28_O_3_S	7.862	39.03
3	Ethanoic acid	C_2_H_4_O_2_	7.896	27.07
4	2-butoxyethyl acetate	C_8_H_16_O_3_	14.298	87.02
5	Butanoic acid, Butyl ester	C_8_H_16_O_2_	14.82	48.01
6	1-hydroxy-2- propanone	C_6_H_6_O	15.96	79.66
7	3,5-bis(1,1-dimethylethyl)phenol	C_14_H_22_O	17.25	83.65
8	6-pentyl-alpha-pyrone	C_10_H_14_O_2_	22.03	67.05
9	Hexadecanoic acid	C_20_H_40_O_2_	22.87	82.69
10	2H-pyran-2-one	C_6_H_10_O_3_	27.65	40.26
11	2,6-dimethyl-naphthalene	C_12_H_12_	29.97	70.81
12	Hexadecane	C_16_H_34_	38.06	82.39
13	2-Octene	C_8_H_16_	49.2	72.7

Note: A total of 13 peaks were obtained and the molecules were identified using GC-MS analysis.

**Table 2:** Antibacterial and antifungal activities of secondary metabolites against bacterial and fungal phytopathogens.

Phytopathogens	MIC (µg/mL)	MBC (µg/mL)
Bacterial phytopathogens		
*X. citri*	63 ± 2.1	82.5 ± 2.5
*X. campestris,*	32 ± 3.2	72.5 ± 1.25
*E. carafavora*	58 ± 3.5	89 ± 5.0
*C. michiganensis*	49 ± 5.5	92 ± 2.5
*A. avenae*	30 ± 2.5	70 ± 1.25
Fungal phytopathogens		
*S. sclerotiorum*	63.5 ± 7.25	120 ± 10.5
*R. solani*	71 ± 3.25	153 ± 2.5
*A. radicina*	58.5 ± 3.0	115.5 ± 1.25
*A. citri*	60.5 ± 5.5	110.2 ± 3.75
*A. dauci*	65 ± 3.75	122.5 ± 3.0

**Table 3:** Extracellular enzyme activities from *T. hamatum* FB10.

Enzymes	Activity (IU/mL)
Chitinase	1.7 ± 0.32
Protease	476 ± 39.6
Polygalacturanase	38.5 ± 2.7
Cellulase	12.9 ± 10.1
Amylase	27.5 ± 2.9
Pectinase	0.2 ± 0.03
Glucanase	58.6 ± 2.2

## Data Availability

Not applicable.
